# Longitudinal effects of a novel advanced pneumatic compression device on patient‐reported outcomes in the management of cancer‐related head and neck lymphedema: A preliminary report

**DOI:** 10.1002/hed.26110

**Published:** 2020-03-18

**Authors:** Carolina Gutiérrez, Harvey N. Mayrovitz, Syed Hassan Shiraz Naqvi, Ron J. Karni

**Affiliations:** ^1^ Department of Physical Medicine & Rehabilitation The University of Texas Health Science Center / McGovern Medical School Houston Texas; ^2^ Department of Otorhinolaryngology ‐ Head & Neck Surgery The University of Texas Health Science Center / McGovern Medical School Houston Texas; ^3^ Division of Physiology, Department of Medical Education Nova Southeastern University, Dr. Kiran C. Patel College of Allopathic Medicine Fort Lauderdale Florida; ^4^ Division of Head & Neck Surgical Oncology, Department of Otorhinolaryngology ‐ Head & Neck Surgery Division of Medical Oncology The University of Texas Health Science Center / McGovern Medical School Houston Texas

**Keywords:** head and neck cancer, head and neck lymphedema, patient‐reported outcomes, pneumatic compression, swallowing difficulty

## Abstract

**Background:**

Head and neck cancer (HNC) survivors experience head and neck lymphedema (HNL), which requires treatment to prevent morbidity. We explore the self‐reported outcomes and satisfaction of patients with HNC receiving treatment for HNL with an advanced pneumatic compression device (APCD).

**Methods:**

HNC survivors (n = 205) prescribed with an at‐home Flexitouch head and neck APCD completed pretreatment and posttreatment self‐reported assessments addressing efficacy, function, and symptoms. Participant average age was 60 years with 74% male. Pre‐post responses for ≥25 days of use were assessed via the non‐parametric Wilcoxon Signed Rank test.

**Results:**

Analysis revealed statistically significant improvement in all symptoms and all function items (*P* < 0.00001). Compliance with prescribed therapy (at least 30 minutes daily) was high with 71% of participants reporting daily use and 87% reporting overall satisfaction.

**Conclusions:**

The reported improvements in function and symptoms, and high compliance rate, provide a rationale for a subsequent randomized controlled trial.

## 1 INTRODUCTION

After completing treatment for head and neck cancer (HNC), patients frequently report having difficulty swallowing, chewing, and breathing, as well as decreased range of motion.^1^, ^2^ This constellation of symptoms has largely been attributed to the effects of surgery and radiation; however, these symptoms are also common in head and neck lymphedema (HNL). HNL develops most commonly as a secondary effect of cancer and its treatment.^3^ Treatments for HNC, including surgery and radiation, disrupt lymphatic structures that are highly concentrated in treated areas and damage surrounding soft tissues thereby increasing the risk for development of lymphedema.^4^, ^5^, ^6^ HNL may also arise as a result of disruption of lymphatic transport by the tumor itself.^3^, ^4^, ^5^

Improved cancer treatments and shifts in disease epidemiology^7^, ^8^, ^9^ have contributed to a recent escalation in the number of HNC survivors, with a corresponding need for additional resources focused on managing the long‐term and late effects of this disease and its treatments.^5^, ^10^, ^11^ Two recent U.S.‐based studies have found that secondary lymphedema occurs in a majority of patients with HNC, with posttreatment prevalence rates of 75% and 90%, respectively^4^, ^5^ Unlike prior reports, these studies used comprehensive lymphedema assessment measures to identify lymphedema occurring internally, externally, and as combined occurrence rates.

HNL typically manifests as clinically evident soft‐tissue swelling in the region affected by HNC treatment.^12^ In contrast to other forms of lymphedema, HNL often occurs directly within treated tissue as well as distally.^12^ Localized accumulation of high‐protein fluid triggers a progressive inflammatory process that often leads to tissue fibrosis, discomfort, disfigurement, functional impairments, and recurrent infections.^10^, ^13^ However, whereas fibrosis is commonly associated with advanced lymphedema in the extremities,^14^ fibrosis often manifests in patients with HNC early in cancer therapy independent of or in conjunction with tissue swelling.^12^ Although acute swelling in patients with HNC may resolve,^6^ lymphedema and/or fibrosis are both present in the vast majority of HNC patients 3‐6 months posttreatment.^5^, ^12^ Over time, their combined effects cause significant tissue changes that make treatment more difficult.^5^, ^12^ HNL may develop internally, in structures such as the larynx and pharynx, and/or externally in the skin and soft tissues of the face and neck.^4^, ^5^, ^6^ Given its distinct characteristics and challenges, HNL requires careful documentation of site‐specific tissue changes and patient‐reported symptoms, as well as modifications to traditional lymphedema management practices.^12^, ^15^

In a study^15^ of over 1200 patients with HNL, more than one third reported functional complaints; most concerning were reports of difficulty swallowing and breathing. These and other functional impairments interfere with activities of daily living and place the patient at risk of malnutrition, dehydration, or additional complications.^10^, ^16^ The severity of functional impairment depends on the proximity of edema to vital anatomic structures and the extent of lymphatic disruption.^17^ The symptom burden of HNL frequently includes decreased range of motion in the neck, musculoskeletal pain, as well as degraded body image and social isolation.^5^, ^16^ The severe impact of HNL on quality of life is well documented.^16^

Research in other cancer populations has shown that early identification and treatment of lymphedema results in improvement of symptoms and reduction in long‐term morbidity.^5^, ^10^ Complete decongestive therapy including manual lymphatic drainage (MLD) massage is the gold standard for treatment of extremity lymphedema,^18^ and evidence supports its use for treatment of HNL. A 2015 study^15^ conducted at a large cancer center of more than 700 patients with HNL secondary to cancer found that 60% of patients who received lymphedema treatment with MLD experienced symptom improvement, regardless of the initial stage or severity of lymphedema. MLD for HNL begins with direction of fluid from the supraclavicular region to the bilateral lymph nodes and progresses to the trunk, neck, and face.^15^, ^19^

To assist patients with self‐management, an advanced pneumatic compression device (APCD) for at‐home use (Flexitouch system; Tactile Medical, Minneapolis, MN) received Food and Drug Administration 510(k) clearance in the summer of 2016 to include the treatment of lymphedema of the head and neck. This physician‐prescribed device is provided to patients for at‐home treatment, with or without therapist‐administered compression and follow‐up. It is contraindicated for patients with active cancer, acute injuries to the skin that may be irritated by stretching or pressing, and a range of symptomatic or uncontrolled cardiovascular or other diseases for which compression or increased circulation would be of concern.

An APCD designed specifically for HNL treatment and well accepted by users may augment current therapies by reducing the significant barriers to self‐care faced by HNC survivors. These barriers include limited use of compression garments, a routine component of lymphedema self‐care that presents unique challenges to HNL patients^20^, ^21^ and can be poorly tolerated^22^; low compliance with therapy^20^; and the high cost of outpatient care.^20^ Outpatient care is likely to place particular burden on the growing number of patients with HNC of working age who have oropharyngeal cancer associated with the human papillomavirus (HPV).^11^ A recent study^17^ that assessed the functional usability of the Flexitouch APCD in treating HNL found that a single 30‐minute, in‐clinic treatment with this device produced clinically and statistically significant reductions in composite measurements of the face (43%) and neck (20%). More than 60% of patients reported feeling better after the single treatment session, whereas 93% reported they would be likely to use this treatment at home. Our current study assesses changes in patient‐reported symptoms and function as well as treatment satisfaction with extended at‐home use of this APCD in patients with cancer‐related HNL.

## 2 MATERIALS AND METHODS

A retrospective analysis was conducted on prospectively gathered survey responses from patients across the United States who were prescribed a Flexitouch system for the at‐home treatment of cancer‐related HNL between October 2016 and September 2017. Patients were required to have a signed consent authorizing use of data for research purposes and to have completed both a pre‐device treatment survey and a follow‐up survey after consistent at‐home device use (25‐day minimum; average 90 days, range 25‐288 days). The study was exempt from IRB oversight by Chesapeake Center for Institutional Review Board Intelligence (CIRBI).

### 2.1 Treatment

**Figure 1:**
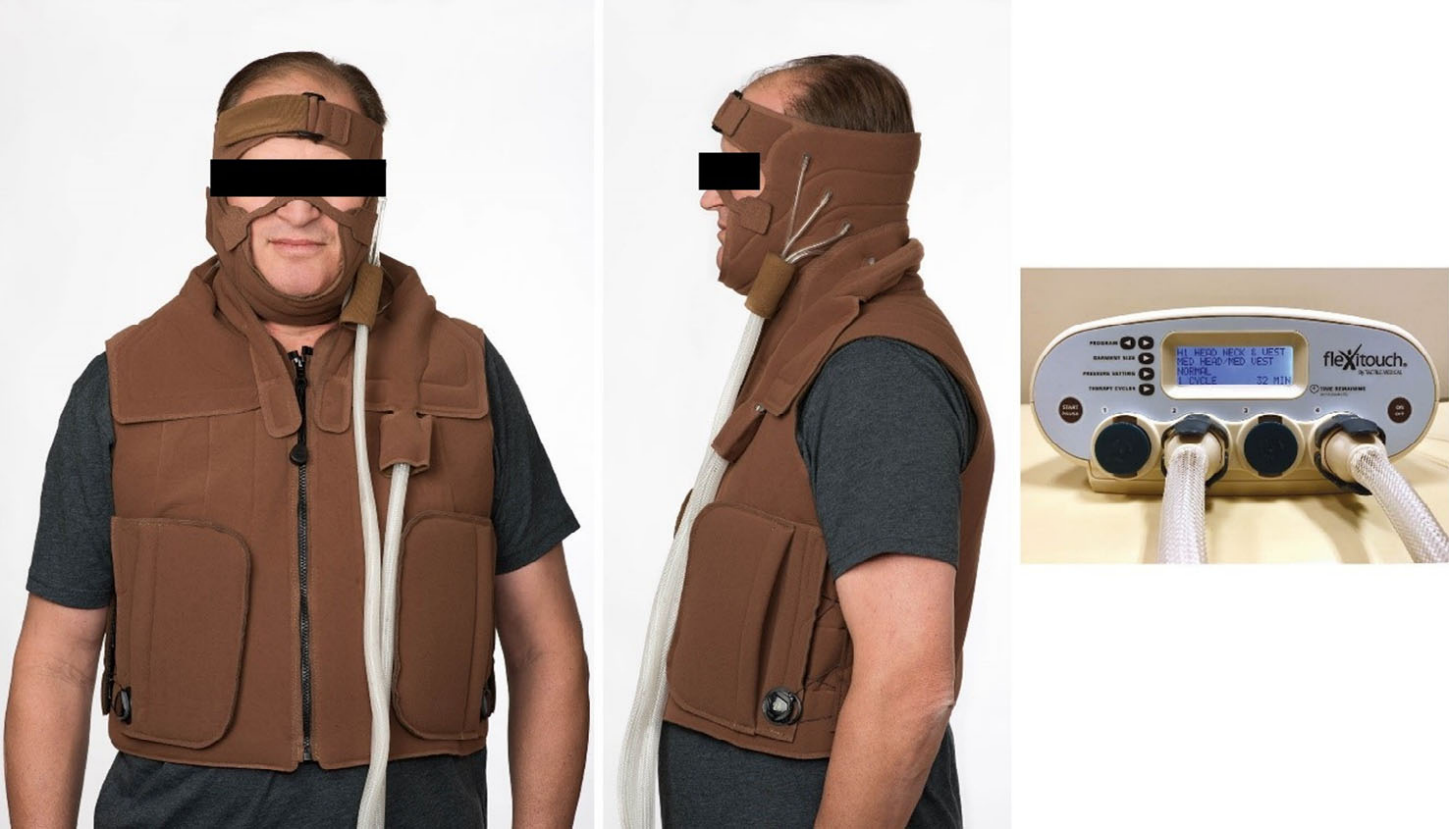
Flexitouch system for head and neck [Color figure can be viewed at wileyonlinelibrary.com]

The Flexitouch system has previously been shown to effectively treat lymphedema in the extremities by stimulating lymphatic function using automated techniques similar to MLD techniques.^23^, ^24^, ^25^, ^26^, ^27^ Garments have been specifically designed to provide treatment to the head and neck using similar, gentle, directional pressure. The Flexitouch system for the head and neck is currently the only FDA‐cleared and available pneumatic compression device for HNC survivors. APCDs (denoted by HCPCS Code E0652) use multi‐chambered garments to deliver adjustable gradient pressure to targeted anatomical sites and can be programmed for pre‐set treatment regimens. The Flexitouch system for head and neck includes a programmable controller paired with inflatable nylon and polyurethane garments with 14 individual chambers that cover parts of the head, neck, and upper torso (Figure 1). The standard 30‐minute program (H1) prescribed for all patients in this study delivers brief applications of dynamic pressure in a wave‐like manner to direct fluid (a) from the neck and the chest toward the axilla, (b) from the head and face toward the neck, and (c) from the head, neck, and face proximally toward the chest lymphatics. Device treatments are pre‐programmed into the controller at Tactile Medical in accordance with the treating clinician's prescription (Table 1). The device does not cover the area of tracheostomy incision, and its effects may vary depending on individual location of healthy lymphatics, limiting its usefulness in some cases. However, given the gravitational advantage of drainage from the upright position of the head,^15^ it is possible that even in such cases the decongestion of surrounding lymphatics may be of some benefit.

**Table 1:** Patient‐reported adherence to prescribed therapy

Q6		
How often are you using the APCD?		
Standard treatment program (H1),^a^ no., (%)		205 (100)
1×/day	Prescribed	177 (86)
	Patient‐reported	109 (53)
2×/day	Prescribed	28 (14)
	Patient‐reported	33 (16)
> 2×/day^b^	Prescribed	0 (0)
	Patient‐reported	3 (2)
3‐6×/week^b^	Prescribed	0 (0)
	Patient‐reported	53 (26)
< 3×/week^b^	Prescribed	0 (0)
	Patient‐reported	7 (3)
Supplementary treatment program (H2),^c^ no. of patients, (%)^d^		99 (48)
Daily	Prescribed	17 (8)
6×/week	Prescribed	1 (<1)
1×/week	Prescribed	1 (<1)
As needed	Prescribed	79 (39)

a 30‐minute primary treatment program aimed at decongesting the head, neck, and chest via incremental proximal clearing of lymphatic fluid in the chest, neck then head followed by delivery of a full head, neck, and chest treatment.

b As part of self‐management, some patients chose to use the device more or less frequently than prescribed.

c 15‐minute supplemental treatment program aimed at decongesting only the head and neck.

d Patient‐reported frequency of use for 1 H2‐programmed ACPD is unknown.

Upon receipt of the device, patients received in‐home instruction from Tactile Medical trainers on device operation per standard company practice. Training included donning/doffing garments, prescribed treatment, and customer care contact information. This training did not include lymphedema care, which is covered by the patient's clinical care team.

### 2.2 Outcome measures

Tactile Medical routinely asks all patients who are prescribed a Flexitouch system to complete a pretreatment survey during their at‐home device training visit and to complete the same survey approximately 1 month after starting treatment. This survey is designed to assess the effects of APCD treatment on symptom control and functional complaints commonly experienced by patients with HNL, including patient‐perceived changes in ability to control lymphedema through at‐home treatment, ability to perform daily activities, level of head and neck pain or discomfort, and difficulty in swallowing or breathing.^15^, ^16^ The survey includes five questions asking patients to rate their symptoms using a Likert scale from 1 to 5, with lower values representing less favorable responses and higher values representing more favorable responses. The posttreatment survey includes five additional questions related to device ease of use, treatment compliance, and overall treatment satisfaction. The data presented in this study were collected from HNL patients in this manner. Survey questions are presented in Table 2.

**Table 2:** Pre‐advanced and post‐advanced pneumatic compression device (APCD) treatment survey questions

Pre‐ and post‐APCD survey questions
How would you describe your ability to control your lymphedema through home treatment? How often has your lymphedema prevented or limited your ability to perform other daily activities? How would you rate your level of head and neck pain or discomfort related to lymphedema? How much difficulty does your head and neck lymphedema cause you when swallowing? How much difficulty does your head and neck lymphedema cause you when breathing?
Post‐APCD only survey questions
6. How often are you using the APCD? 7. Rate your overall satisfaction with the APCD. 8. How easy are the APCD garments to put on, use, and take off? 9. How comfortable is the treatment provided by your APCD? 10. How do you feel after a treatment session?

Patients who did not return the follow‐up questions were contacted by phone and asked to complete the survey during an interview with Tactile Medical clinical services personnel. Additional demographic information and aspects of medical history, including type of cancer and cancer treatment, were obtained through review of the medical record.

### 2.3 Data analysis

All responses to survey questions were ranked from 1 to 5 with pre‐survey to post‐survey analyses conducted via the non‐parametric Wilcoxon Signed Rank test for each of the posed questions to determine the statistical significance of pre‐to‐post changes. The survey was designed so that in all cases higher numbers represented better or improved conditions. Because there were five symptom questions, a *P*‐value deemed to represent a statistically significant change was a priori set to *P* < 0.01. For the additional five compliance and satisfaction questions, patient responses were analyzed and their frequency distribution was characterized for each question. Results are generally represented as mean ± SD except where specifically noted.

## 3 RESULTS

A total of 499 eligible patients received the head and neck APCD during the study period, with 239 patients completing both a pretreatment and posttreatment survey (48% response rate). Of those, 232 (97%) patients had used the device for at least 4 weeks. Seven participants were excluded due to having HNL that was unrelated to HNC. Twenty patients were excluded from the pre‐to‐post analysis due to missing survey responses. The final study population included 205 patients with HNC‐related HNL. Patients were predominantly male (152, 74%) with a mean age of 60 (range 13‐83), the majority having squamous cell carcinoma. The most common tumor sites were the oropharynx and oral cavity, accounting for 67% of patients studied. More than half the study patients (59%) received combined modality cancer treatment, which included primary tumor resection and radiotherapy. Payers frequently require patients to have tried and failed conservative therapy before receiving an APCD; thus the majority of patients had received CDT and/or used compression garments or bandaging for 4 to greater than 8 weeks before use of the APCD (75% and 62%, respectively). Nearly half (48%) of this patient population initiated APCD use within 6 months of HNL diagnosis; other patients initiated use 6 months to 1 year (34%), 2 to 5 years (11%), or greater than 5 years (7%) after diagnosis. The average duration of APCD use at the time of post‐APCD treatment survey completion was 90 days (range 25‐288). A summary of patient demographics and characteristics is provided in Table 3.

**Table 3:** Patient demographics and characteristics

Characteristics	No. of patients = 205
Age median, (range)	60 (13‐83)
Male sex, no. (%)	152 (74)
Disease specific	
HNC tumor site, no. (%)	
Oropharynx	72 (35)
Oral cavity	65 (32)
Larynx	25 (12)
Other^a^	14 (7)
Unknown^b^	14 (7)
Thyroid	11 (5)
Salivary gland	4 (<2)
HNC cancer treatment	
Primary HNC treatment, no. (%)	
Surgery,^c^ radiation, LND,^d^ and chemotherapy	67 (33)
Radiation and chemotherapy	44 (21)
Surgery, radiation, and LND	36 (17)
Surgery and LND	12 (6)
Surgery and radiation	10 (5)
Surgery, radiation, and chemotherapy	9 (4)
Surgery and chemotherapy	9 (4)
Radiation only	8 (4)
Surgery only	5 (2)
Radiation and LND	3 (1)
Radiation, LND, and chemotherapy	2 (<1)
Conservative lymphedema therapy before APCD use	
Duration of complete decongestive therapy, no. (%)	
No answer/blank	45 (22)
None	2 (<1)
1‐3 weeks	5 (2)
4‐8 weeks	84 (41)
8+ weeks	69 (34)
Duration of compression garments/bandaging treatment, no. (%)	
Blank/NA	46 (22)
None	19 (9)
1‐3 weeks	12 (6)
4‐8 weeks	70 (34)
>8 weeks	58 (28)
APCD lymphedema therapy	
Time of initiation of APCD therapy post‐lymphedema diagnosis, no. (%)	
<6 months	98 (48)
6 months to 12 years	69 (34)
2‐5 years	23 (11)
>5 years	15 (<7)
Duration of APCD use at posttreatment survey, mean, (range)	90 (25‐288)

a Includes nasopharynx, hypopharynx, melanoma, basal cell, esophagus, Hodgkin's lymphoma, and non‐Hodgkin's lymphoma.

b Sqaumous cell carcinoma of the head and neck; primary tumor site not provided.

c Surgery = primary tumor resection.

d Lymph node dissection (LND).

The pre‐to‐post APCD symptom question responses demonstrated statistically significant improvements in all of the five queried symptom questions as summarized in Table 4.

**Table 4:** Pretreatment to posttreatment comparisons

	Q1		Q2		Q3		Q4		Q5	
	How would you describe your ability to control lymphedema through home treatments?		How often has your lymphedema prevented or limited your ability to perform other daily activities?		How would you rate your level of head and neck pain or discomfort related to lymphedema?		How much difficulty does your head and neck lymphedema cause you when swallowing?		How much difficulty does your head and neck lymphedema cause you when breathing?	
Duration of lymphedema	*pre‐tx*	*post‐tx*	*pre‐tx*	*post‐tx*	*pre‐tx*	*post‐tx*	*pre‐tx*	*post‐tx*	*pre‐tx*	*post‐tx*
<0.5 years										
Mean	1.93	3.65	3.44	4.09	3.24	3.76	3.09	3.68	4.21	4.49
SD	1.01	0.93	1.36	1.16	1.15	0.93	1.26	1.11	1.03	0.93
No. of patients	95	95	95	95	95	95	95	95	95	95
*P*‐value	<0.001		<0.001		<0.001		<0.001		<0.01	
Effect size	1.77		0.52		0.50		0.50		0.29	
0.5‐2 years										
Mean	1.82	3.61	2.99	3.96	3.19	3.62	2.70	3.46	3.69	4.40
SD	0.86	1.01	1.36	1.21	1.18	1.07	1.26	1.26	1.20	0.81
No. of patients	72	72	72	72	72	72	72	72	72	72
*P*‐value	<0.001		<0.001		<0.01		<0.001		<0.001	
Effect size	1.91		0.75		0.38		0.60		0.71	
2‐5 years										
Mean	2.08	3.54	3.46	4.12	2.83	3.54	3.17	3.75	3.83	4.46
SD	1.11	1.02	1.38	1.03	1.13	0.98	1.31	1.33	1.13	0.88
No. of patients	24	24	24	24	24	24	24	24	24	24
*P*‐value	<0.001		<0.05		<0.01		<0.05		<0.001	
Effect size	1.37		0.55		0.67		0.44		0.63	
>5 years										
Mean	1.71	3.36	2.57	3.64	2.57	2.64	2.50	3.00	3.64	4.29
SD	0.91	0.93	1.34	1.28	0.94	1.15	1.29	1.36	1.15	0.99
No. of patients	14	14	14	14	14	14	14	14	14	14
*P*‐value	<0.01		<0.05		0.763		0.205		0.075	
Effect size	1.79		0.82		0.07		0.38		0.61	
Total										
Mean	1.89	3.61	3.22	4.01	3.13	3.61	2.90	3.57	3.94	4.44
SD	0.96	0.96	1.38	1.17	1.16	1.03	1.28	1.21	1.13	0.88
No. of patients										
*P*‐value	<0.00001*		<0.00001*		<0.00001*		<0.00001*		<0.00001*	
	1 = Poor 2 = Fair 3 = Good 4 = Very good 5 = Excellent		1 = Always 2 = Often 3 = Sometimes 4 = Rarely 5 = Never		1 = Severe 2 = Somewhat severe 3 = Moderate 4 = Mild 5 = None					

* Statistically significant.

Expressed as pre vs post responses there was a positive shift reported in the ability to control lymphedema symptoms through at‐home treatment (1.89 ± 0.96 vs 3.61 ± 0.96; *P* < 0.00001). There was also a decrease in how often the participant's lymphedema prevented or limited their ability to perform daily activities (3.22 ± 1.38 vs 4.01 ± 1.17; *P* < 0.00001). Participants also reported improvement in the level of head and neck pain or discomfort (3.13 ± 1.16 vs 3.61 ± 1.03; *P* < 0.00001) and decreased difficulty with swallowing (2.90 ± 1.28 vs 3.57 ± 1.21; *P* < 0.00001) as well as improved ability to breathe (3.94 ± 1.13 vs 4.44 ± 0.88; *P* < 0.00001).

Survey responses indicated overall consistent head and neck APCD use, with 71% of patients using it at least once a day, another 26% using the device three to six times per week (Table 1). Eighty‐seven percent of patients indicated they were “satisfied” or “very satisfied” with head and neck APCD therapy. More than 80% of patients found the treatment to be “comfortable” or “very comfortable” and nearly 90% reported feeling better after therapy. The majority of patients (78%) found the device “easy” or “very easy” to use (Table 5).

**Table 5:** Patient satisfaction with APCD therapy

	Q7		Q8		Q9		Q10	
	Rate your overall satisfaction with the APCD		How easy are your APCD garments to put on, use and take off?		How comfortable is the treatment provided by your APCD?		How do you feel after a treatment session?	
	Response, no., (%)		Response, no., (%)		Response, no., (%)		Response, no., (%)	
	1 = Very dissatisfied	4 (1.9)	1 = Very difficult	1 (0.5)	1 = Very uncomfortable	2 (1.0)	1 = Much worse	0 (0.0)
	2 = Dissatisfied	3 (1.5)	2 = Difficult	10 (4.9)	2 = Uncomfortable	2 (1.0)	2 = Somewhat worse	2 (1.0)
	3 = Neutral	20 (9.7)	3 = Neutral	34 (16.5)	3 = Neutral	34 (16.5)	3 = No change	21 (10.2)
	4 = Satisfied	81 (39.3)	4 = Easy	81 (39.3)	4 = Comfortable	103 (50.0)	4 = Somewhat better	109 (53.4)
	5 = Very satisfied	98 (47.6)	5 = Very easy	80 (38.8)	5 = Very comfortable	65 (31.6)	5 = Much better	73 (35.4)
Avg	4.29		4.11		4.11		4.23	
*SD*	0.85		0.88		0.88		0.67	

## 4 DISCUSSION

**Figure 2:**
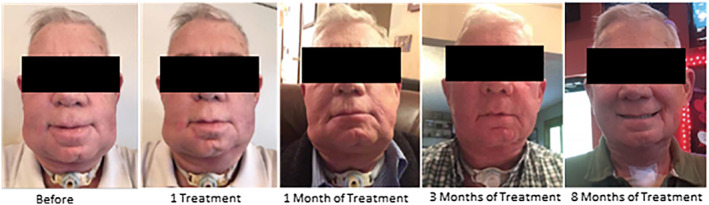
PT127593 response to Flexitouch treatment [Color figure can be viewed at wileyonlinelibrary.com]

This investigation provides a first look at patient‐reported outcomes, compliance, and overall satisfaction in a large cohort after multiple at‐home treatments. These results reveal statistically significant, positive effects over time on patient‐reported ability to control lymphedema, perform activities of daily living, and reduce lymphedema‐related pain and difficulties with swallowing and breathing. The results also document high rates of satisfaction with the device that may have contributed to patient‐reported symptom improvement and compliance (Figure 2). Seventy‐one percent of patients were fully compliant with prescribed daily 30‐minute APCD therapy, whereas 26% used the APCD at least three to six times per week. Larger prospective studies with a control group are needed to evaluate effects of treatment in conjunction with complete decongestive therapy, determine optimum duration of the treatment, and determine durability of the treatment effects.

Observational findings suggest that patients whose APCD therapy did not begin until late after HNL diagnosis (>5 years) experienced less improvement in pain reduction and swallowing difficulties, although small patient populations in these cohorts prevented statistical significance. These observational findings would seem to align with previous reports^5^, ^12^ that prolonged HNL/fibrosis may cause significant tissue changes that diminish treatment response. As HNL remains widely unrecognized and under‐addressed,^28^ further study of therapeutic practices for late‐stage HNL patients is warranted.

There are several limitations to this study. Primarily, the lack of a control group does not allow for conclusions on efficacy. However, the study met its primary aim of exploring patient‐reported outcomes and satisfaction with the Flexitouch system for home use. The survey results will be used to inform the design of a future randomized trial. As an observational study, additional limitations include wide variation in the time interval (25‐288 days) between the pretreatment and posttreatment assessments. Finally, data were collected via a Tactile Medical customer survey and not a validated patient‐reported outcomes (PRO) questionnaire. Although this questionnaire provides valuable insight into patient satisfaction and device usability, use of a validated survey would strengthen future prospective studies. Strengths include the large number of patients included in the analysis.

## 5 CONCLUSIONS

Studied parameters, such as ability to perform activities of daily living and functional improvements in swallowing and breathing, demonstrated statistically significant positive changes from pre‐ to post‐device use. Our findings suggest the potential utility of at‐home use of this device in contributing to improved quality of life in this patient population and provide a rationale for a subsequent randomized controlled trial to objectively assess improvement in symptoms with the use of a head and neck APCD.

## CONFLICT OF INTEREST

Ron J. Karni, MD, serves on the Scientific Advisory Board and Speakers Bureau for Tactile Medical. Harvey N. Mayrovitz, PhD, is a scientific consultant to Tactile Medical.
